# Host-Associated and Environmental Microbiota of Hatchery-Reared Sichuan Taimen (*Hucho bleekeri*): Community Structure and Functional Profiling

**DOI:** 10.3390/ani16132089

**Published:** 2026-07-06

**Authors:** Qinyao Wei, Yeyu Chen, Huanchao Yang, Jun Du, Hua Li, Zhaobin Song

**Affiliations:** 1 Key Laboratory of Bio-Resources and Eco-Environment of the Ministry of Education, College of Life Sciences, Sichuan University, Chengdu 610065, China; 2 Fisheries Research Institute, Sichuan Academy of Agricultural Sciences, Chengdu 611730, China; 3 Observation and Research Station of Sichuan Province of Fish Resources and Environment in Upper Reaches of the Yangtze River, Sichuan Academy of Agricultural Sciences, Chengdu 611730, China; 4 Key Laboratory of Sichuan Province for Fishes Conservation and Utilization in the Upper Reaches of the Yangtze River, Chengdu 611730, China

**Keywords:** *Hucho bleekeri*, host-associated microbiota, metagenomics, artificial rearing

## Abstract

The diversity and complexity of symbiotic microbiota in fish may significantly influence the host’s physiological, metabolic and immunological functions. In order to understand the microbial assembly in Sichuan taimen (*Hucho bleekeri*), an endangered fish species in the upper reaches of the Yangtze River, the microbiota of the skin, oral cavity and feces of artificially reared individuals and the microbiota of the rearing water were characterized through metagenomic sequencing. The results demonstrated that Pseudomonadota were shared across the skin, oral cavity, feces and rearing water, suggesting that they may constitute a shared microbial group connecting the aquatic environment and host mucosal surfaces. Based on functional prediction analyses, these taxa were potentially associated with organic matter degradation, nutrient cycling, and microbial and immune homeostasis. Likewise, Actinomycetota and Bacillota were consistently detected across multiple mucosal tissues and were predicted to be associated with nutrient transformation, antimicrobial defense, and the maintenance of mucosal microbial stability. Fusobacteriota were detected solely in feces, suggesting a strong tissue-specific colonization capacity. The alpha diversity of the microbiota did not differ significantly among tissues, and the beta diversity revealed strong clustering of host-associated samples and clear separation from water samples. Functional annotation further revealed that the water microbiota exhibited broader yet more dispersed functional potential, whereas host-associated microbiota showed stronger functional specialization closely aligned with host physiological demands. Collectively, the findings are better presented as baseline information for future comparative and hypothesis-driven studies in Sichuan taimen.

## Simple Summary

Fish live in close association with many microscopic organisms that inhabit their skin, mouth, digestive system, and the surrounding water. These microbial communities may play important roles in fish health, nutrition, and disease resistance. In this study, we investigated shared microbial communities and their predicted functions in the skin, oral cavity, feces, and rearing water of the endangered Sichuan taimen (*Hucho bleekeri*) under artificial culture conditions. High-throughput sequencing revealed that the fish and the surrounding water shared several dominant microbial taxa, while some microorganisms were specific to particular body sites. Community structure analyses demonstrated a clear separation between fish-associated and water-associated microbial communities. Functional predictions indicated that microorganisms in the rearing water possessed a broader range of potential functions, whereas fish-associated microbiota exhibited greater stability and functional specialization. Overall, this study provides baseline information on the microbial communities of *H. bleekeri* under hatchery conditions, improves our understanding of host–environment microbial interactions, and offers scientific support for the conservation, health management, and optimization of artificial propagation practices for this endangered species.

## 1. Introduction

Microbial communities exhibit complex structures and play a vital role in supporting the health and welfare of aquaculture species [1,2]. These microorganisms inhabit various mucosal surfaces of fish and exist in the surrounding water. Real-time and accurate monitoring of their dynamic changes is essential for deciphering host–microbe interactions within the holobiont, which directly affects the productivity and sustainability of aquaculture systems [3,4,5,6].

The microbiota of fish across different tissues and organs (e.g., skin, gills, and gut) differ significantly [7]. The skin serves as the first line of defense against pathogenic invasion in vertebrates and hosts a vast number of microorganisms on its surface. Unlike mammalian skin, teleost fish skin is characterized by abundant mucus-secreting cells and a living epidermis that remains in direct contact with the surrounding aquatic environment [8]. These microorganisms form close interactions with the host and play essential roles in growth promotion, nutrient absorption, development, and resistance to pathogenic infections [9]. A study on rainbow trout (*Oncorhynchus mykiss*) revealed that, among the five examined anatomical sites—skin, gills, olfactory rosette, anterior intestine, and posterior intestine—the skin exhibited the highest microbial diversity. Notably, approximately 50% of the skin-associated microbial diversity was localized within the epithelial cell layer, suggesting that live epithelial cells may provide a more favorable environment for microbial colonization [10].

Similarly, the oral cavity microbiome plays a crucial role in fish health, as microbial dysbiosis in this region has been linked to disease onset. For example, disturbances in the oral microbial community of Atlantic salmon (*Salmo salar*) have been associated with the development of yellow mouth disease [11]. Moreover, the gut microbiota plays multiple roles in the healthy growth of fish. It contributes to nutrient utilization by degrading complex polysaccharides and producing vitamins and short-chain fatty acids, thereby supplying essential energy sources [12]. In addition, it modulates immune maturation, maintains immune homeostasis, protects against pathogen invasion, and preserves intestinal ecological stability through competitive exclusion of harmful microorganisms [13]. The aquatic environment also plays a crucial role in shaping microbial communities. Microorganisms in aquaculture water are vital for nutrient cycling and maintaining ecosystem function, directly impacting the health of cultured fish [14,15]. Sustaining microbial abundance and diversity in the aquatic environment supports the well-being of cultured fish [16].

Microorganisms in the aquatic environment exhibit complex interrelationships and dynamic interactions. The skin microbiota of fish primarily originate from the surrounding water, sediments, and the surfaces of other aquatic organisms. These microbes adhere to the skin through contact with suspended particles and interactions within the aquatic ecosystem. Notably, fish can selectively recruit beneficial microbes, thereby forming a distinct and host-specific skin microbial community [17]. For example, fish skin has been shown to support selective microbial colonization that acts as a biological barrier against aquatic pathogens [18]. In contrast, microbes from the water and diet entering the gastrointestinal tract must undergo physiological and biochemical adaptation to the gut environment, ultimately establishing a stable and host-adapted intestinal microbiota [19]. Despite these findings, it remains unclear whether microbial communities inhabiting the skin, oral cavity, feces, and the surrounding water environment share a common microbiota. Moreover, the functional roles of these shared microbes and the relative contribution of each niche to overall fish health are still not fully understood.

Sichuan taimen (*Hucho bleekeri*) is an endemic and rare species only distributed in the upper reaches of the Yangtze River [20]. Due to the extreme scarcity of wild populations, *H. bleekeri* has been listed as first-class national protected animal in China (National Forestry and Grassland Administration & Ministry of Agriculture and Rural Affairs of P. R China, announcement No. 3) and critically endangered on the International Union for Conservation of Nature (IUCN) Red List of Threatened Species [21]. At present, artificial domestication, propagation, and release comprise important conservation efforts for this species. To systematically evaluate the growth performance and health status of *H. bleekeri* under captive rearing conditions, it is necessary to conduct in-depth investigations of their symbiotic microbial communities. In this study, metagenomic sequencing was employed to characterize the microbial composition and functional differences in the skin, oral cavity, feces, and rearing water of *H. bleekeri*, with the aim of identifying shared microbiota under aquaculture conditions and providing insights to support improved feeding and management practices. Such studies present baseline microbiome information for hatchery-reared fish from a single environment. These data will support future studies on artificial rearing effects, post-release adaptability and conservation management.

## 2. Materials and Methods

### 2.1. Sample Collection

Skin mucus, oral mucus and feces of broodstock *H. bleekeri* and water from the rearing pond were collected from the Jiguanshan Base of the Fisheries Research Institute, Sichuan Academy of Agricultural Sciences, in March 2025. Skin and oral mucus samples were collected by swabbing the lateral surface of the skin and oral cavity of each fish, respectively. Each sampling site was swabbed two to three times, and two to three swabs per fish were pooled into a single preservation tube. The abdomen in front of the fish cloaca was gently pressed to expel feces, which were then collected using sterile swabs. To minimize potential contamination, the cloacal area was thoroughly cleaned with sterile saline before sampling. The swabs were then immediately transferred into sterile 15 mL tubes prefilled with DNA preservation solution. Throughout the sampling process, sterile techniques were strictly followed, and a new pair of sterile gloves and sterilized sampling tools was used for each individual fish to prevent cross-contamination from skin mucus and environmental sources. Water samples (1.5 L) from the rearing ponds of broodstock *H. bleekeri* were filtered through 0.45 μm membrane filter units, with each filter used for a single sample. After filtration, the filter cartridges were transferred directly into 15 mL centrifuge tubes.

Skin mucus, oral mucus, and fecal samples were collected from eight individual *H. bleekeri*. The experimental fish were 7–8 years old, including four females and four males, with body lengths of 72.00 ± 11.31 cm and body weights of 6.22 ± 3.02 kg. Fish were fed live prey (*Siniperca chuatsi* and *Carassius auratus*) at 2–3-day intervals and were fasted for approximately one week prior to sampling. The stocking density was approximately 1 kg/m^3^ in a single rearing pond (60 m^3^). The aquaculture system was maintained at 5–6 °C, without the use of antibiotics or probiotics, and was disinfected with salt every two weeks. All fish were clinically healthy at the time of sampling. Due to sample collection constraints and DNA quality filtering, the final dataset included skin mucus (*n* = 6), oral mucus (*n* = 4), and fecal samples (*n* = 7). All fish were reared in a single aquaculture pond under identical conditions. Three water samples were collected from different locations within the pond to characterize the microbial community of the rearing water. Downstream analyses were conducted at the sample level to characterize community-wide microbiome patterns, without incorporating individual identity as a grouping factor in statistical models. All animal experiments were approved by the Ethics Committee of the College of Life Sciences, Sichuan University (Approval No.: SCU251011001).

### 2.2. DNA Extraction, Quantification, and Metagenomic Sequencing

Microbial genomic DNA was extracted using the CTAB method. Briefly, samples were lysed in CTAB buffer with lysozyme at 65 °C. The lysate was subjected to phenol: chloroform: alcohol (25:24:1) extraction followed by chloroform: alcohol (24:1) purification. DNA was precipitated with isopropanol at −20 °C, washed with 75% ethanol, air-dried, and dissolved in ddH_2_O. RNA was removed by RNase A treatment at 37 °C for 15 min. A high-quality DNA sample (OD260/280 = 1.8~2.0, OD260/230 ≥ 2.0) was used to construct the sequencing library. Genomic DNA (1 μg) from each sample was randomly fragmented to approximately 350 bp using a Covaris ultrasonicator (Covaris, Inc., Woburn, MA, USA) for library preparation. Sequencing libraries were constructed through end repair, A-tailing, adapter ligation, purification, and PCR amplification following standard protocols. After library construction, fragment size distribution and library integrity were assessed using AATI, and libraries meeting the expected size distribution were retained. Library concentration was accurately quantified by qPCR (effective concentration > 3 nM) to ensure sequencing quality. Qualified libraries were pooled according to their effective concentrations and the required sequencing depth and sequenced on an Illumina NovaSeq platform with paired-end 150 bp (PE150) reads. Raw sequencing reads were processed using fastp to obtain high-quality clean data for downstream analysis. The filtering criteria were as follows: (i) reads containing adapter sequences were removed; (ii) reads in which more than 50% of bases had a quality score ≤ 5 were discarded; and (iii) reads containing more than 10% ambiguous bases (N) were removed. Clean reads were assembled using MEGAHIT (version 1.2.9) under the meta-large preset mode. Assembly parameters were set to --end-to-end, --sensitive, -I 200, and -X 400 [22,23]. The resulting scaffolds were subsequently split at N positions to generate scaftigs without ambiguous bases [24,25]. DNA extraction, library preparation, and sequencing were all performed by Novogene Co., Ltd. (Beijing, China). To assess the potential contribution of host-derived sequences to the “Others” category, host-read filtering was evaluated on representative samples using Bowtie2 (version 2.5.4).

### 2.3. Gene Prediction and Abundance Analysis

Open reading frames (ORFs) were predicted from assembled contigs using MetaGeneMark (version 2.1), a widely used tool for identifying protein-coding sequences in metagenomic data. To reduce redundancy and construct a comprehensive non-redundant gene catalog, all predicted genes were clustered using CD-HIT (version 4.5.8) with default parameters, thereby removing highly similar sequences and retaining representative genes [26,27]. Subsequently, quality-filtered clean reads from each sample were mapped back to the non-redundant gene catalog using Bowtie2 (version 2.5.4). Gene abundance was calculated based on the number of reads aligning with each gene [24,28]. This approach generated a quantitative profile of gene content and abundance across all samples, providing the foundation for downstream functional annotation and comparative analyses. The comprehensive details on all software parameters, database versions, and access dates are provided in Table S1.

### 2.4. Microbial Taxonomic Annotation

Taxonomic annotation of microbial unigenes was performed using DIAMOND (version 2.1.9) by aligning sequences against the Micro_NR database, which was constructed from bacterial, fungal, archaeal, and viral sequences extracted from the NCBI NR database [29]. For each unigene, alignments with an E-value ≤ 1 × 10^−5^ were retained. Because a single unigene may produce multiple significant matches, taxonomic assignments were determined using the Lowest Common Ancestor (LCA) algorithm implemented in MEGAN based on the retained hits [30]. Based on the LCA annotation results and the gene abundance table, taxonomic abundance profiles and gene-count tables were generated for each sample at different taxonomic ranks (kingdom, phylum, class, order, family, genus, and species). The abundance of a given taxon in a sample was calculated as the sum of the abundances of all genes assigned to that taxon, whereas the gene count for a given taxon was defined as the number of annotated genes with non-zero abundance in that sample [28,31]. Given the high proportion of sequences assigned to “Others”, resulting from both low-abundance taxa and taxonomically unresolved reads under the DIAMOND–MEGAN LCA framework, taxonomic analyses were focused on the phylum and class levels, where taxonomic assignments are relatively more stable and less sensitive to annotation uncertainty.

### 2.5. Functional Annotation

Functional annotation of unigenes was performed by aligning sequences against multiple reference databases. For the annotation of metabolic and signaling pathways, sequences were aligned to the Kyoto Encyclopedia of Genes and Genomes (KEGG) using DIAMOND (version 2.1.9). Orthologous group classification and broad functional prediction were performed using the evolutionary genealogy of genes: Non-supervised Orthologous Groups (eggNOG) database. Carbohydrate-active enzymes (CAZymes) were identified by mapping metagenomic sequences to the Carbohydrate-Active enZYmes (CAZy) database, whereas antibiotic-resistance genes (ARGs) were annotated using established resistance-gene databases, including the comprehensive antibiotic-resistance database (CARD) and Resfams. For both functional categories, the relative abundance of each category was calculated as the sum of the relative abundances of all genes assigned to that category [28,31]. ARGs were further classified according to resistance mechanisms and taxonomically assigned to microbial phyla based on metagenomic annotations. Only ARGs associated with phyla shared across all four sample groups were retained for downstream visualization and comparative analyses.

### 2.6. Statistical Analysis

Alpha diversity was assessed using the Chao1, Shannon, and Simpson indices to evaluate microbial richness and evenness within samples. These indices were calculated in R (version 4.3.3) and visualized using GraphPad Prism (version 8.0). The non-parametric Kruskal–Wallis H test was used to assess statistical significance. Means that do not share a common letter are significantly different (*p* < 0.05).

Beta diversity was analyzed to compare microbial community composition among samples. Multivariate ordination analyses, including principal component analysis (PCA), principal coordinates analysis (PCoA), and non-metric multidimensional scaling (NMDS), were performed to visualize differences in community structure. PCA was conducted on normalized and scaled abundance data using R (version 4.3.3). PCoA and NMDS were based on Bray–Curtis dissimilarity matrices calculated using the vegan package. NMDS was performed with two dimensions using the metaMDS function, and stress values < 0.2 were considered indicative of a reliable ordination. To statistically evaluate differences in community composition among groups, permutational multivariate analysis of variance (PERMANOVA) was performed using the adonis2 function in the vegan package with 999 permutations. Pairwise comparisons between groups were conducted using the pairwise Adonis package (available at https://github.com/pmartinezarbizu/pairwiseAdonis, accessed on 22 June 2026), and *p*-values were adjusted using the Benjamini–Hochberg method to control for multiple testing. Functional beta diversity analyses based on KEGG annotations were performed using Bray–Curtis dissimilarity and visualized by PCoA. Differential KEGG pathway analysis was conducted using the Linear Discriminant Analysis Effect Size (LEfSe) method based on KEGG Orthology (KO) abundance profiles, with *p* < 0.05 and an LDA score > 3 considered statistically significant.

## 3. Results

### 3.1. Taxonomic Composition and Diversity of the Microbial Communities

**Figure 1:**
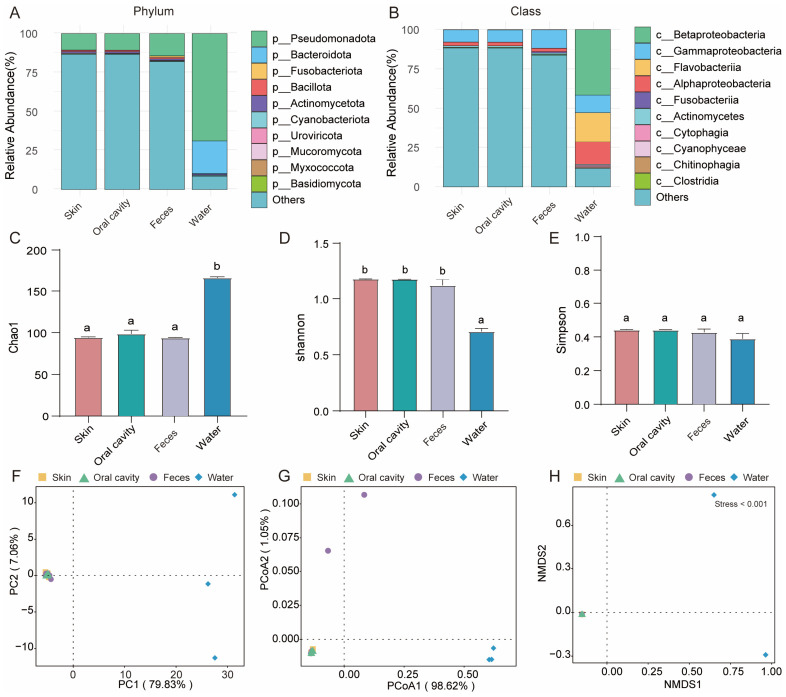
Taxonomic composition and diversity of microbial communities of skin, oral cavity, feces and rearing water of *H. bleekeri*. (**A**,**B**) Relative abundance of the top 10 taxa at the phylum and class levels. (**C**–**E**) Alpha diversity indices. (**F**–**H**) PCA, PCoA, and NMDS analyses of microbial community structure.

After excluding the “Other” category, Pseudomonadota consistently represented the most abundant phylum across all sample groups (Figure 1A), with average relative abundances of 10.49%, 10.51%, 14.21%, and 68.50% in the skin, oral cavity, feces, and water, respectively (Figure 1A, Table S3). Pseudomonadota, Actinomycetota, and Bacillota consistently ranked as the top three shared microbial phyla across the skin, oral cavity, and feces (Figure S1). At the class level, Betaproteobacteria (41.41%) were the dominant microbial in water. The relative abundances of Gammaproteobacteria in the skin, oral cavity, feces and water were 7.73%, 7.74%, 11.62%, and 11.21%, respectively (Figure 1B, Table S4). Fusobacteria (0.89%) were only enriched in the feces (Table S4).

Alpha diversity analysis revealed that water samples exhibited the highest Chao1 richness index among all sample types, whereas the Shannon diversity index was significantly lower in water (Figure 1C,D, *p* < 0.05, Table S5). In contrast, no significant differences were observed in the Simpson diversity index among the four sample types (Figure 1E, *p* > 0.05). Consistently, PCA, PCoA, and NMDS analyses demonstrated that microbial communities from the skin, oral cavity, and feces clustered closely together, whereas water-associated communities formed a distinct and separate group (Figure 1F–H). These patterns were statistically supported by PERMANOVA based on Bray–Curtis dissimilarities (F = 139.70, R^2^ = 0.963, *p* = 0.001) (Figure S2A,B).

### 3.2. Abundance Analysis of KEGG Functional Genes

**Figure 2:**
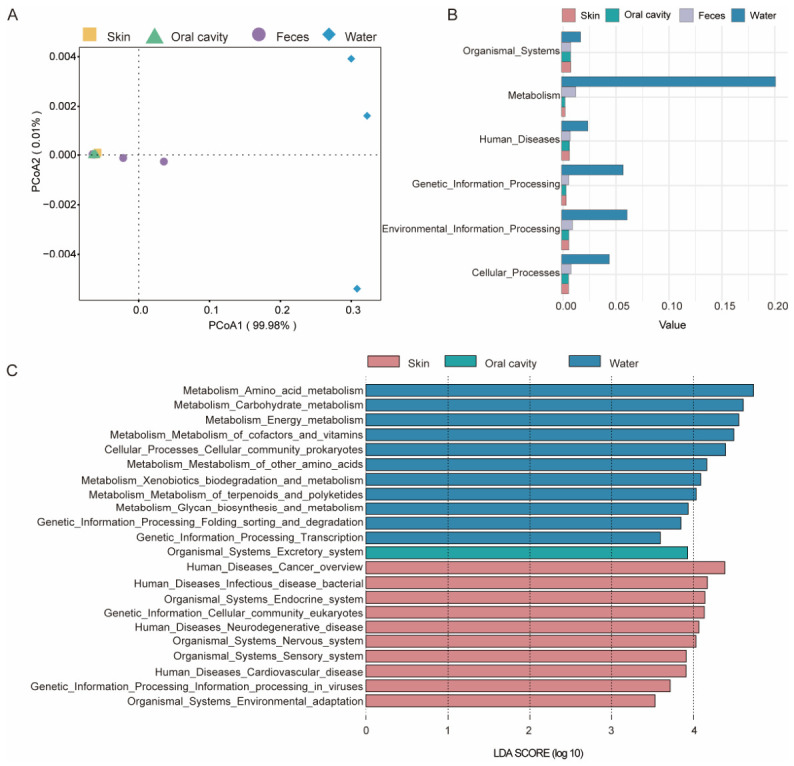
Functional profiles of microbial communities from skin, oral cavity, feces and water. (**A**) PCoA illustrating differences in predicted microbial functions. (**B**) KEGG functional enrichment analysis of microbial communities. (**C**) LDA score distribution of KEGG pathways identified by LEfSe analysis.

The functional prediction was performed using the KEGG database to explore the functional characteristics of symbiotic and water environment microbiota. PCoA revealed that the microbial functional profiles of the water samples were significantly distinct from those of the skin, oral cavity and feces (Figure 2A), which was statistically confirmed by PERMANOVA (F = 200.85, R^2^ = 0.974, *p* = 0.001) (Figure S2C). Comparison analysis of KEGG pathway enrichment showed that organismal systems, metabolism, genetic information processing, environmental information processing, and cellular processes were more prominently represented in the water compared to the skin, oral cavity and feces (Figure 2B). LEfSe analysis of KEGG metabolic pathways revealed that the excretory system was significantly enriched in the oral cavity (Level 2, under organismal systems, Level 1) (Figure 2C). The skin displayed significant differences in organismal systems and genetic information processing (Level 1) (Figure 2C). In the water, significant differences were observed in metabolism, cellular processes, and genetic information processing (Level 1) (Figure 2C). Notably, no significantly enriched biomarkers were identified in the feces group by LEfSe analysis under the predefined significance criteria. Therefore, feces-associated biomarkers were not reported in the results.

### 3.3. Analysis of EggNOG Composition

**Figure 3:**
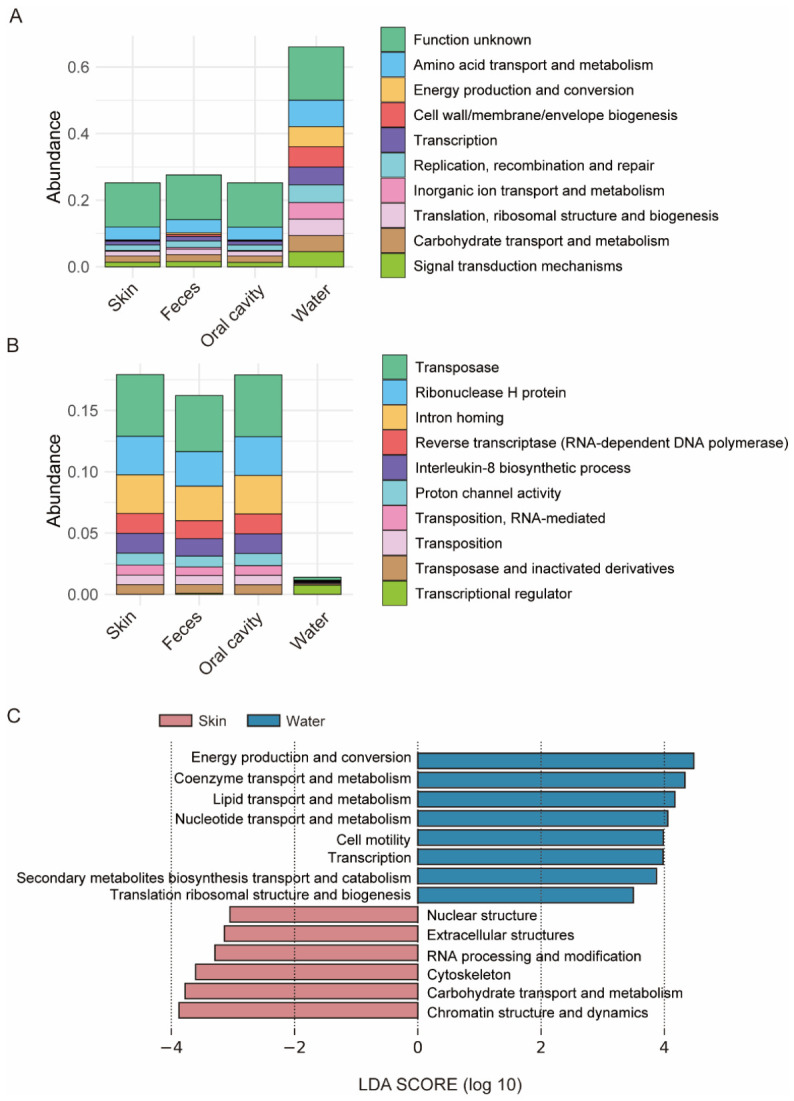
EggNOG functional diversity analysis. (**A**,**B**) Relative abundance statistics of functional gene categories at Level 1 and Level 2 annotated by the eggNOG database. (**C**) LDA score distribution histogram at Level 1.

The eggNOG analysis showed that at Level 1, functional genes were generally more abundant in water, while the relative proportions of genes involved in amino acid transport and metabolism and carbohydrate transport and metabolism were similar across the skin, oral cavity, feces, and water (Figure 3A). At Level 2, transcriptional regulator functions were most enriched in the water, whereas the skin, oral cavity, and feces exhibited higher numbers of enriched functions overall (Figure 3B). LEfSe analysis indicated that significant differences were observed only between the skin and water. Specifically, the skin was significantly enriched in genes associated with nuclear structure, extracellular structures, RNA processing and modification, cytoskeleton, carbohydrate transport and metabolism, and chromatin structure and dynamics (Figure 3C). Notably, several enriched functional categories, such as nuclear structure and chromatin structure and dynamics, are generally associated with eukaryotic systems and should be interpreted cautiously.

### 3.4. Analysis of CAZy Composition

**Figure 4:**
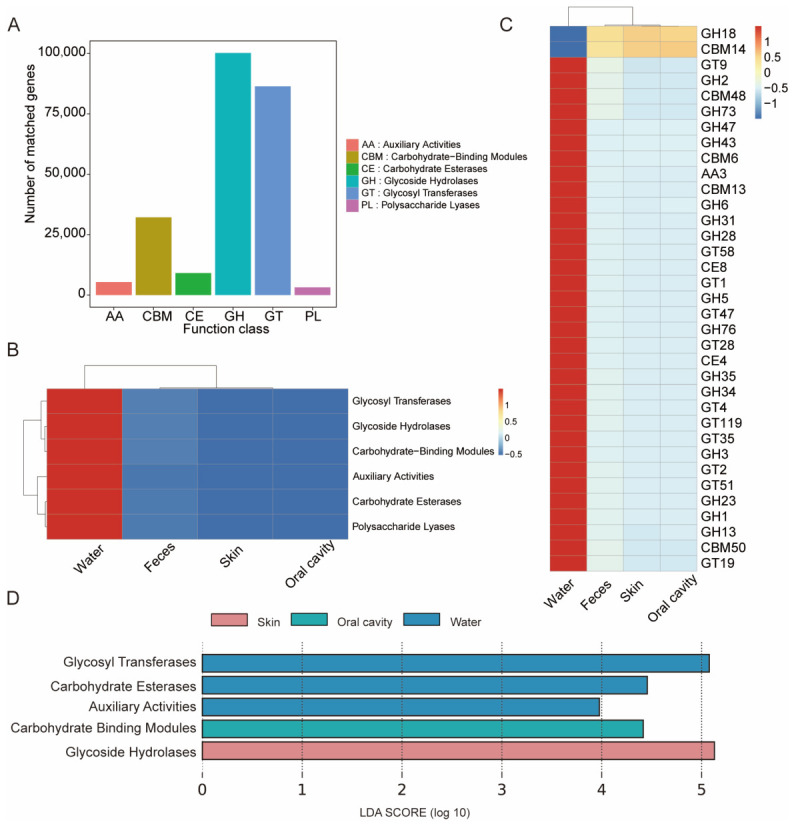
CAZy functional diversity analysis. (**A**) Statistics of gene annotations based on the CAZy database. (**B**,**C**) Heatmaps of CAZy annotations at Level 1 and Level 2. (**D**) LDA score distribution histogram at Level 1.

The statistical chart of annotated gene numbers at Level 1 in the CAZy database showed that glycoside hydrolases (GHs) had the highest enrichment (Figure 4A). The Level 1 heatmap showed a higher relative abundance of carbohydrate-related genes in the rearing water compared with the oral cavity, skin, and feces (Figure 4B). At Level 2 of the CAZy database, GH18 and CBM14 were more abundant in the oral cavity, skin, and feces than in the rearing water (Figure 4C). LEfSe analysis indicated that significant differences were observed only among the skin, oral cavity, and rearing water. Specifically, glycoside hydrolases were significantly enriched in the skin, carbohydrate-binding modules in the oral cavity, and glycosyl transferases, carbohydrate esterases, and auxiliary activities in the rearing water (Figure 4D).

### 3.5. Predicted Antibiotic-Resistance Genes

**Figure 5:**
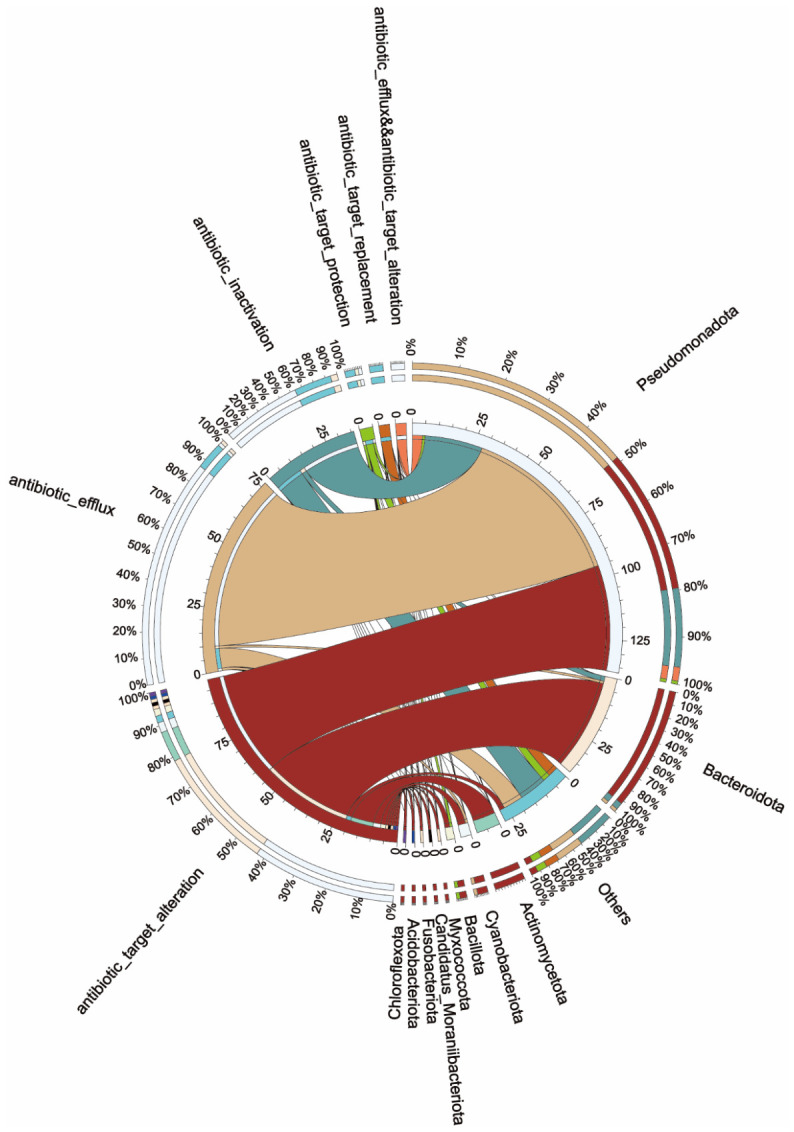
This circular plot depicts the associations between microbial phyla and antibiotic-resistance mechanisms. The right side of the outer ring represents microbial taxa, whereas the left side represents resistance mechanism categories. Sector length corresponds to the total number of ARGs, outer percentages indicate the proportional composition within each category, and the inner scale reflects absolute counts. Ribbons connecting sectors illustrate the links between phyla and resistance mechanisms, with ribbon width proportional to the number of ARGs of a given mechanism within each phylum. Ribbon colors differentiate source phyla or resistance mechanism categories.

The circular plot illustrates the associations between microbial phyla and ARGs in the metagenomic dataset (Figure 5). At the phylum level, ARG-like sequences were predominantly assigned to Pseudomonadota across different resistance mechanism categories, including antibiotic efflux, antibiotic inactivation, and antibiotic target alteration. Bacteroidota and Actinomycetota also contributed annotated ARG-like sequences, while other phyla accounted for relatively fewer assignments. In addition, resistance mechanisms such as target protection and other minor categories were also observed at lower annotation frequencies.

At the ARG family level, diverse resistance-gene categories were identified based on database annotation, including tetracycline resistance genes (e.g., tet(A), tet(C), tet(M), tet(Q), and tet(X)), sulfonamide resistance genes (sul1 and sul2), β-lactam–associated genes (including OXA-type and FOX-type β-lactamase homologs), and macrolide resistance genes (e.g., mphA, mphE, and EreD). Aminoglycoside resistance–associated genes (e.g., AAC, ANT, and APH families), as well as multidrug efflux-related genes (e.g., adeF), were also detected across the samples (Table S6). It should be noted that a large proportion of sequences (>99.8%) were not classified as known ARGs in the database (Non-ARO), indicating that most reads could not be assigned to established resistance-gene categories (Table S6). Therefore, these results should be interpreted as annotation-based descriptive profiles rather than direct evidence of functional resistance activity or selection pressure.

## 4. Discussion

This study provides an ecological overview of microbial communities associated with different host niches and the surrounding aquatic environment, emphasizing high-level taxonomic patterns and functional potential rather than fine-scale taxonomic resolution. The microbiome, defined as the community of microorganisms associated with a host, plays a critical role in host growth, development, and survival [32]. Accordingly, comprehensive characterization of the fish-associated microbiome is essential for understanding their ecological roles within the host and their interactions with the aquatic environment [33]. In the present study, both shared and niche-specific microbial communities were identified across skin, oral cavity, feces and rearing water of *H. bleekeri*.

Pseudomonadota was identified as the top the most abundant phyla shared across the skin, oral cavity, feces and water. Previous research has demonstrated that Pseudomonadota predominantly inhabit cold-water environments [34], which aligns well with the cold-water adaptation characteristics of *H. bleekeri*. In general, the skin and oral cavity are directly exposed to the rearing water, and their associated microbiota are thought to partially mirror the microbial diversity in the ambient aquatic environment [35,36]. Pseudomonadota is the microbial group with the highest relative abundance (excluding the “Other” category) in the feces of Sichuan taimen, which is consistent with the reported studies [37,38]. The dominance of Pseudomonadota may reflect their broad ecological adaptability and metabolic versatility, as suggested in previous studies.

Pseudomonadota and Bacteroidota exhibited relatively high abundances in the rearing water samples. This finding is consistent with previous results on the microbial communities of aquaculture pond water for Sichuan taimen cultured at different altitudes [16], confirming that Pseudomonadota consistently accounts for a high proportion of the bacteria in aquaculture ponds. The higher enrichment of Bacteroidota observed in the Maerkang aquaculture ponds may be attributed to the higher stocking density at this site [16]. These environments are rich in proteins, polysaccharides, and glycoproteins, which constitute preferred substrates for Bacteroidota [39].

Bacillota and Actinomycetota are widely recognized for their capacity to produce a variety of antimicrobial compounds, a trait that ecologically favors their colonization of mucus-rich frontline immune barriers of the host, such as the skin and oral cavity where mucus secretion is abundant [40,41]. These antimicrobial compounds inhibit the establishment of potential pathogenic microorganisms and thus contribute to host defense against pathogens. In the present study, Bacillota and Actinomycetota exhibited higher relative abundances in the skin and oral cavity compared with rearing water, which is consistent with the ecological functions of the skin and oral cavity as important immune barriers.

Pseudomonadota, Bacillota, Fusobacteriota, and Bacteroidota are the predominant phyla in the gut microbiota of most marine and freshwater fish [7,42]. In the present study, Fusobacteriota was predominantly detected in feces, which is consistent with previous reports describing its frequent occurrence in the intestinal microbiota of carnivorous teleost, indicating that its abundance is closely associated with fish feeding habits [33,43]. Consistently, Xu et al. (2026) confirmed that Fusobacteria (class level) showed the lowest gut abundance in herbivorous *Schizothorax* among species with different feeding habits [44]. Members of this phylum are well adapted to protein-rich diets and capable of utilizing amino acids and other protein-derived substrates, which is consistent with the carnivorous feeding habits of *H. bleekeri* [45].

Alpha and beta diversities are key metrics used to assess the complexity and organization of community structure [46]. In this study, no significant differences were identified in the alpha diversity of the microbiota among the skin, oral cavity, and feces. However, the alpha diversity of the microbiota in all of those significantly differed from that of the rearing water. Beta diversity analysis further revealed that the fish-associated microbial communities clustered closely together and were clearly separated from those of the rearing water, indicating distinct microbial community structures between host-associated and environmental samples. KEGG-based PCoA showed a clear functional separation between water- and fish-associated tissues, suggesting that host–environment differentiation of microbiota occurs not only in composition but also in function. EggNOG analysis indicated that water samples had higher overall functional abundance at Level 1 but lower abundance at Level 2, indicating differences in functional category distribution patterns between environmental and host-associated microbiota. CAZy annotation further revealed differences in the abundance of carbohydrate-active enzymes between water and host-associated microbiota. These differences reflect distinct functional compositions across habitats, while the ecological mechanisms driving these patterns remain to be determined [7,47,48].

Although significant differences between host-associated microbiota and rearing water were detected, the relatively small number of water samples (*n* = 3) represents a limitation of the present study. Small sample sizes can reduce the statistical power of alpha diversity, beta diversity, and differential abundance analyses, potentially limiting the detection of subtle community differences. In addition, the unequal sample sizes among groups may further affect the robustness of community comparisons. Consequently, the absence of significant differences among some sample groups should not necessarily be interpreted as evidence of biological similarity. Furthermore, the lack of a wild control group and sampling restricted to a single season limit the generalizability of the observed patterns across different environmental conditions and natural population variability. This study was primarily conducted as an exploratory assessment to describe the potential antibiotic-resistance-gene profiles within the captive rearing environment and to complement the overall characterization of the microbial community, rather than to infer direct effects of antibiotic exposure or management practices.

Collectively, our results reveal a close ecological association between host-associated and environmental microbiota of *H. bleekeri* under artificial rearing conditions. It should be noted that the individuals examined in this study were derived from cultured systems, where rearing environments, water quality, and dietary composition differ to some extent from natural habitats and may influence the structure and function of symbiotic microbial communities of the species. Future studies incorporating comparisons with wild individuals will help to systematically elucidate the compositional and functional differences in symbiotic microbiota across contrasting habitats, thereby providing a basis for future investigations into the potential relationships between microbiota composition and fish physiological performance in aquaculture systems.

## 5. Conclusions

This study employed metagenomic sequencing to investigate the composition, diversity, and functional potential of commensal microbial communities in the skin, oral cavity, feces, and water of *H. bleekeri* under artificial rearing conditions. The results showed clear differences between host-associated and rearing water microbiota in both community composition and functional profiles. Host-associated samples exhibited distinct microbial assemblages compared with environmental water samples. Pseudomonadota were consistently detected across both host-associated and environmental samples, while Actinomycetota and Bacillota were frequently observed across multiple mucosal tissues, indicating shared microbial components among host-associated habitats. Fusobacteriota were detected only in feces samples, showing a distinct distribution pattern among tissues. Alpha diversity did not differ significantly among host-associated tissues, whereas beta diversity analysis revealed clear separation between host-associated and water microbiota. Functionally, water microbiota exhibited different distributions of functional categories, while host-associated microbiota displayed distinct functional compositions. Overall, this study provides baseline information on the microbiota of hatchery-reared *H. bleekeri*. These findings may contribute to future studies exploring host–microbe–environment interactions and their potential implications for aquaculture systems; however, experimental validation will be required to confirm these hypotheses.

## Data Availability

Metagenomic sequencing raw data have been deposited in the NCBI Sequence Read Archive (SRA) under Bio-Project Number PRJNA1473018. The accession numbers of the raw sequencing data are provided in Table S2.

## Supplementary Materials

The following supporting information can be downloaded at https://www.mdpi.com/article/10.3390/ani16132089/s1, Figure S1: Taxonomic composition across skin, oral cavity and feces; Figure S2: Beta-diversity analyses based on Bray–Curtis dissimilarity; Table S1: List of analysis software; Table S2: Information of metagenomic sequencing; Table S3: Microbial composition of skin, oral cavity, feces and rearing water at phylum level; Table S4: Microbial composition of skin, oral cavity, feces and rearing water at class level; Table S5: Alpha diversity indices at phylum level; Table S6: Relative abundance of antibiotic resistance genes annotated based on the Antibiotic Resistance Ontology (ARO).
