# Supplementary material for: Host-Associated and Environmental Microbiota of Hatchery-Reared Sichuan Taimen (Hucho bleekeri): Community Structure and Functional Profiling
Source: Animals (Basel). 2026 Jul 6;16(13):2089. doi: 10.3390/ani16132089 (PMC13359490; doi:10.3390/ani16132089)
Supplement: Supplementary file 1 [file animals-16-02089-s001.zip › animals-4381035-supplementary.pdf]

Supplementary Manuscript

Host-Associated and Environmental Microbiota of Hatchery-Reared Sichuan Taimen (*Hucho bleekeri*): Community Structure and Functional Profiling

Qinyao Wei[1, 2], Yeyu Chen[2, 3], Huanchao Yang[2, 3], Jun Du[2, 3], Hua Li[2, 3, 4, *] and Zhaobin Song[1, *]

[1] Key Laboratory of Bio-Resources and Eco-Environment of the Ministry of Education, College of Life Sciences, Sichuan University, Chengdu, 610065, China

[2] Fisheries Research Institute, Sichuan Academy of Agricultural Sciences, Chengdu, 611730, China

[3] Observation and Research Station of Sichuan Province of Fish Resources and Environment in Upper Reaches of the Yangtze River, Sichuan Academy of Agricultural Sciences, Chengdu, 611730, China

[4] Key Laboratory of Sichuan Province for Fishes Conservation and Utilization in the Upper Reaches of the Yangtze River, Chengdu, 611730, China

* Correspondence: lihua1982@scsaas.cn; zbsong@scu.edu.cn

Table S1. List of analysis software.

Analysis conttent	Software	Version	Parameters	Access date
Data quality control	fastp	0.23.1	-g -q 5 -u 50 -n 15 -l 150 --overlap_diff_limit 1 --overlap_diff_percent_limit 10	2025.3.20-2025.4.1
Assembly	megahit	1.2.9	--presets meta-large -m 180000000000 -t 2	2025.3.20-2025.4.1
Gene prediction	GeneMark.hmm	2.1	-a -d -f G -p 1	2025.3.20-2025.4.1
Gene de-redundancy	CD-HIT	4.5.8	-T 6 -G 0 -aS 0.9 -g 1 -d 0 -c 0.95 -n 5 -M 8000	2025.3.20-2025.4.1
Reads mapping	bowtie2	2.5.4	--end-to-end --sensitive --no-hd --no-sq -I 200 -X 400 --threads 8	2025.3.20-2025.4.1
Heatmap	R	2.15.3	R package = corrplot	2025.3.20-2025.4.1
Micro-NR database alignment and annotation	diamond	2.1.9	-p 4 -e 1e-5 -k 50	2025.3.20-2025.4.1
KEGG alignment and annotation	diamond	2.1.9	-p 4 -e 1e-5 -k 50 --id 30 --sensitive	2025.3.20-2025.4.1
eggNOG alignment and annotation	diamond	2.1.9	-p 4 -e 1e-5 -k 50 --id 30 --sensitive	2025.3.20-2025.4.1
CAZy alignment and annotation	diamond	2.1.9	-p 4 -e 1e-5 -k 50 --id 30 --sensitive	2025.3.20-2025.4.1

Table S2. Information of metagenomic sequencing.

SampleID	Accession	Raw_Base(G)	Clean_Base(G)	Clean_Q20(%)	Clean_Q30(%)	Clean_GC(%)	Effective (%)
FS1	SRR38965822	16.63	15.68	98.52	94.97	42.32	94.29
FS2	SRR38965821	13.74	13.04	98.55	95.04	42.31	94.89
FS3	SRR38965810	25.46	23.94	98.38	94.71	42.38	94.04
MS1	SRR38965809	12.11	11.36	98.38	94.71	42.23	93.73
MS2	SRR38965808	15.07	14.31	98.55	95.07	42.33	94.90
MS3	SRR38965807	16.96	16.06	98.56	95.06	42.10	94.67
FF1	SRR38965806	14.62	14.03	98.74	95.43	44.36	95.98
FF2	SRR38965805	15.49	14.52	98.40	94.72	42.03	93.68
FF3	SRR38965804	14.91	13.97	98.36	94.54	41.93	93.72
MF1	SRR38965803	19.82	18.73	98.45	94.79	42.49	94.48
MF2	SRR38965820	14.35	13.62	98.58	95.15	42.73	94.88
MF3	SRR38965819	13.06	12.17	98.28	94.39	41.76	93.15
MF4	SRR38965818	15.03	14.16	98.49	94.86	41.92	94.19
MM1	SRR38965817	13.08	12.35	98.45	94.91	42.11	94.41
MM2	SRR38965816	13.34	12.53	98.44	94.72	41.84	93.88
MM3	SRR38965815	14.70	13.83	98.40	94.79	42.16	94.03
MM4	SRR38965814	15.76	14.88	98.46	94.84	42.49	94.45
W1	SRR38965813	15.27	15.13	98.95	95.51	56.44	99.10
W2	SRR38965812	17.88	17.72	99.05	95.94	54.61	99.13
W3	SRR38965811	18.09	17.89	99.09	96.13	53.32	98.89

Notes: FS: Female skin; MS: Male skin; FF: Female feces; MF: Male feces; MM: Male oral cavity; W: water.

Table S3. Microbial composition of skin, oral cavity, feces and rearing water at phylum level.

Phylum level	Skin	Oral cavity	Feces	Water
Others	86.83%	86.81%	82.22%	8.60%
Pseudomonadota	10.49%	10.51%	14.21%	68.50%
Bacteroidota	0.13%	0.14%	0.10%	21.29%
Fusobacteriota	0.00%	0.00%	0.90%	0.00%
Bacillota	0.87%	0.87%	0.91%	0.10%
Actinomycetota	0.96%	0.96%	0.89%	0.68%
Cyanobacteriota	0.02%	0.02%	0.02%	0.55%
Uroviricota	0.00%	0.00%	0.10%	0.07%
Mucoromycota	0.41%	0.40%	0.38%	0.03%
Myxococcota	0.00%	0.00%	0.00%	0.16%
Basidiomycota	0.29%	0.28%	0.26%	0.02%

Table S4. Microbial composition of skin, oral cavity, feces and rearing water at class level.

Class level	Skin	Oral cavity	Feces	Water
Others	88.40%	88.36%	83.83%	11.79%
Betaproteobacteria	0.10%	0.12%	0.12%	41.41%
Gammaproteobacteria	7.73%	7.74%	11.62%	11.21%
Flavobacteria	0.06%	0.08%	0.05%	18.52%
Alphaproteobacteria	2.30%	2.29%	2.12%	14.59%
Fusobacteria	0.00%	0.00%	0.89%	0.00%
Actinomycetes	0.96%	0.96%	0.89%	0.61%
Cytophagia	0.01%	0.01%	0.01%	0.80%
Cyanophyceae	0.02%	0.02%	0.02%	0.49%
Chitinophagia	0.02%	0.02%	0.02%	0.54%
Clostridia	0.39%	0.38%	0.43%	0.04%

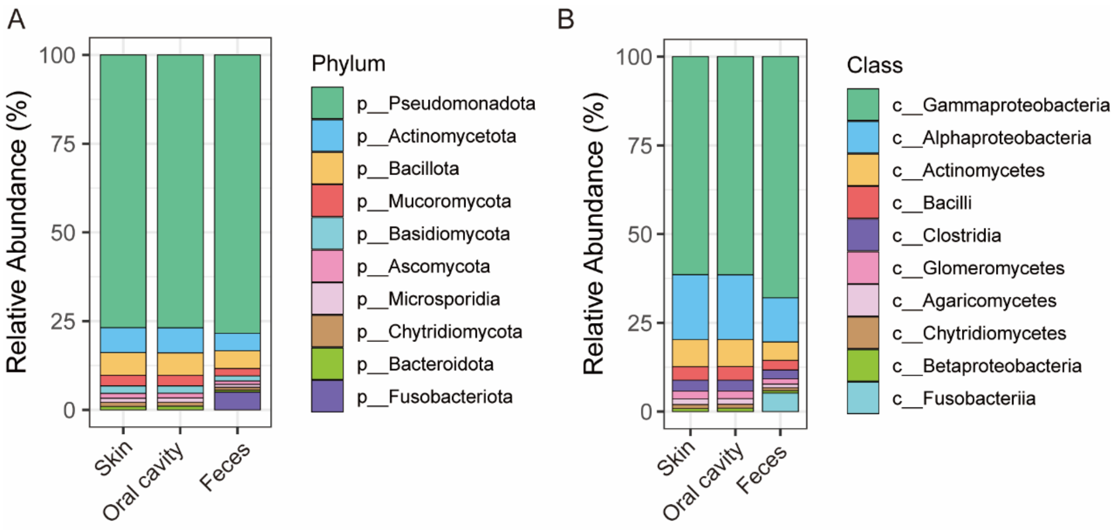

Figure S1. Taxonomic composition across skin, oral cavity and feces. (A-B) Microbial composition of the top 10 taxa at the phylum and class levels in skin, oral cavity, and feces.

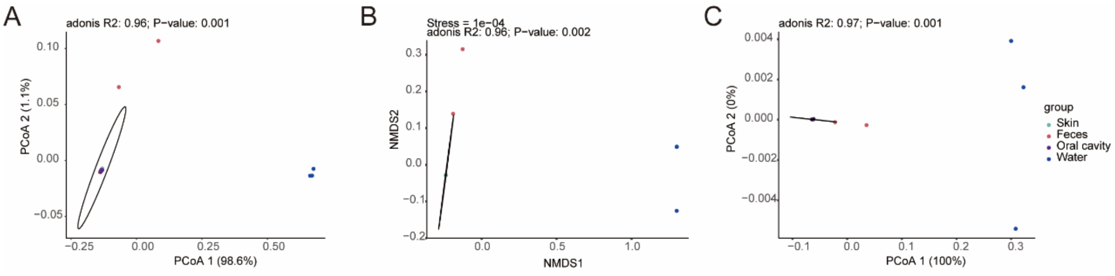

Figure S2. Beta-diversity analyses based on Bray–Curtis dissimilarity. (A–B) PCoA and NMDS ordinations of microbial communities at the phylum level, with corresponding PERMANOVA results. (C) PCoA ordination of KEGG Level 1 functional profiles, with corresponding PERMANOVA results.

Table S5. Alpha diversity indices at phylum level.

sample	ACE	chao1	Shannon	Simpson	observed_species	goods_coverage
FS1	94.49176	94.5	1.176347	0.442393	94	1
FS2	96.25786	96	1.180639	0.443846	96	1
FS3	94	94	1.17275	0.441473	94	1
MS1	93	93	1.169659	0.440244	93	1
MS2	96.24972	96	1.174958	0.441788	96	1
MS3	94	94	1.191243	0.448553	94	1
FF1	94.22077	94	0.815869	0.320997	94	1
FF2	95	95	1.172625	0.442164	95	1
FF3	94.30351	94	1.172194	0.441403	94	1
MF1	95	95	1.173217	0.439726	95	1
MF2	93	93	1.164913	0.470253	93	1
MF3	93.66429	93.33333	1.171917	0.441225	93	1
MF4	94.2552	94	1.174986	0.442955	94	1
MM1	93	93	1.175333	0.4419	93	1
MM2	95.41869	96	1.178956	0.443686	95	1
MM3	95.16822	95	1.170664	0.440962	95	1
MM4	105.5547	111.5	1.180269	0.44328	101	1
W1	167.2055	167	0.658118	0.330691	167	1
W3	168.82	168.2	0.736697	0.427628	167	1
W2	165.1844	165	0.733523	0.412824	165	1

Table S6. Relative abundance of antibiotic resistance genes annotated based on the Antibiotic Resistance Ontology (ARO).

ARO_name	Skin	Feces	Oral cavity	Water
QnrS2	2.45E-08	0.000146	0	3.51E-07
adeF	1.61E-08	1.03E-05	3.56E-08	5.05E-05
vanT_gene_in_vanG_cluster	2.01E-08	0	4.38E-08	5.11E-05
OXA-912	3.33E-09	7.52E-06	0	9.77E-08
qacG	0	0	0	1.79E-05
MCR-4.3	0	2.36E-06	0	0
vanY_gene_in_vanB_cluster	0	2.32E-06	0	2.65E-06
cphA3	0	3.04E-06	0	6.34E-08
rsmA	3.46E-08	2.20E-06	1.80E-08	6.03E-06
vanY_gene_in_vanM_cluster	0	0	5.58E-	1.09E-05

			09	
vanH_gene_in_vanO_cluster	0	3.46E-06	0	0
vanW_gene_in_vanB_cluster	0	0	0	3.94E-06
Acinetobacter_baumannii_AbaQ	3.16E-09	0	3.46E-09	2.61E-06
FosA8	4.11E-08	1.10E-06	0	2.63E-06
vanW_gene_in_vanG_cluster	1.75E-08	1.86E-08	2.64E-08	2.52E-06
vanY_gene_in_vanA_cluster	0	0	0	2.96E-06
Pseudomonas_aeruginosa_soxR	5.29E-09	0	0	2.46E-06
vanH_gene_in_vanA_cluster	1.55E-09	0	8.28E-09	2.12E-06
Tet (C)	0	2.19E-09	0	2.21E-06
qacJ	0	0	0	2.40E-06
sul1	6.10E-09	3.18E-07	8.94E-09	2.19E-06
vanG	0	0	7.48E-09	1.30E-06

AAC (6')-Ib9	0	2.11E-07	0	1.88E-06
FOX-16	0	3.21E-07	0	7.15E-08
ANT (3")-IIa	0	3.75E-07	0	8.65E-07
OXA-551	0	2.48E-07	0	2.57E-08
FosI	0	0	0	1.05E-06
vanY_gene_in_vanF_cluster	0	0	0	8.30E-07
TRU-1	0	2.32E-07	0	9.54E-09
vanW_gene_in_vanI_cluster	2.32E-08	2.73E-08	0	5.71E-07
aadA5	0	2.56E-09	0	7.91E-07
Mycobacteriumtuberculosis_rpsL_mutations_conferring_resistance_to_Streptomycin	0	0	0	5.37E-07
aadS	0	0	1.06E-08	8.53E-07
AAC (6')-32	0	0	0	7.03E-07
floR	4.72E-09	2.00E-07	7.39E-09	7.53E-07
OXA-10	0	1.74E-07	0	6.93E-08
cmlA5	0	1.61E	0	7.43E-09

		-07		
QnrVC4	0	1.56E-07	0	0
vanH_gene_in_vanB_cluster	2.90E-09	0	0	7.86E-07
Tet (A)	0	3.10E-08	0	7.48E-07
sul2	3.24E-09	6.62E-08	4.56E-09	7.74E-07
catB3	0	1.39E-07	0	1.08E-07
Tet (X)	6.59E-09	1.88E-09	3.47E-09	6.02E-07
APH (6)-Id	2.05E-08	3.27E-08	0	6.41E-07
cmlA9	0	0	3.06E-09	6.38E-07
dfrA14	0	1.62E-07	0	0
APH (3")-Ib	1.06E-08	7.08E-08	0	6.65E-07
EreD	0	0	0	2.91E-07
dfrA1	6.29	1.12E	0	0

	E-09	-07		
LnuH	0	0	0	4.79E-07
FosXCC	0	0	0	1.89E-07
Mrx	0	9.25E-08	0	1.29E-08
qacL	0	3.32E-08	0	1.59E-07
tet(Q)	5.76E-08	0	0	1.98E-07
mphE	3.37E-09	0	1.16E-08	1.75E-07
mphA	0	7.20E-08	0	0
tet(M)	0	0	0	1.73E-07
NonARO	1	0.999818	1	0.999817507
